# Association between Platelet Parameters and Glaucoma Severity in Primary Open-Angle Glaucoma

**DOI:** 10.1155/2019/3425023

**Published:** 2019-05-09

**Authors:** Yi Ma, Jianping Han, Shengjie Li, Aiping Zhang, Wenjun Cao, Xinghuai Sun

**Affiliations:** ^1^Department of Clinical Laboratory, Eye & ENT Hospital, Shanghai Medical College, Fudan University, Shanghai 200031, China; ^2^Department of Ophthalmology & Visual Science, Eye & ENT Hospital, Shanghai Medical College, Fudan University, Shanghai 200031, China; ^3^State Key Laboratory of Medical Neurobiology, Institutes of Brain Science and Collaborative Innovation Center for Brain Science, Fudan University, Shanghai 200032, China; ^4^NHC Key Laboratory of Myopia, Key Laboratory of Myopia, Chinese Academy of Medical Sciences, Shanghai Key Laboratory of Visual Impairment and Restoration, Fudan University, Shanghai 200031, China

## Abstract

**Purpose:**

To evaluate platelet parameters in primary open-angle glaucoma (POAG) patients and to explore the association between platelet parameters and POAG severity.

**Methods:**

A total of 402 consecutive POAG patients and 408 healthy control subjects from the Department of Ophthalmology and Visual Science, Eye and ENT Hospital, Fudan University, were consecutively recruited between January 2016 and October 2018. Detailed ophthalmological and systematic examinations were performed. Blood samples for platelet parameters, including platelet count (PLT), platelet distribution width (PDW), plateletcrit (PCT), mean platelet volume (MPV), and platelet large cell ratio (P-LCR), were analyzed using an automated hematology analyzer in the department of clinical laboratory science of the hospital. The POAG subgroups were classified according to age (<50, ≥50), gender, and visual field mean deviation (MD): mild (MD ≤ 6 dB), moderate (6 dB < MD ≤ 12 dB), and severe (MD > 12 dB).

**Results:**

In POAG patients, PLT counts (207.08 ± 54.70 *∗* 10^9^/L) were significantly lower (*p*=0.001) than those of the control group (220.46 ± 55.85 *∗* 10^9^/L). In the POAG group, PDW (13.76 ± 3.16 fL) and MPV (10.46 ± 1.32 fL) values were significantly higher (all *p* < 0.001) than those of the control group (PDW 11.82 ± 2.44 fL, MPV 10.13 ± 1.10 fL). PDW and MPV values were highest in the severe POAG group (PDW 14.49 ± 2.99 fL; MPV 10.74 ± 1.39 fL), followed by the moderate group (PDW 12.50 ± 3.14 fL; MPV 10.02 ± 1.08 fL) and then the mild group (PDW 11.82 ± 2.44 fL; MPV 9.92 ± 0.76 fL), with statistically significant differences observed between mild-severe POAG and moderate-severe POAG groups by LSD post hoc test. Multiple linear regression analyses showed a significant association between PDW and MD (*β* = 0.430, *p* < 0.001) and MPV and MD (*β* = 0.363, *p*=0.001). Logistic regression analyses revealed that PDW (OR = 1.297, 95% CI = 1.011–1.663) was associated with the severity of POAG.

**Conclusions:**

PDW and MPV values were significantly elevated in POAG patients, and PDW was positively associated with disease severity, which suggested the possibility that platelet activation be involved in pathomechanisms of POAG.

## 1. Introduction

Glaucoma is a heterogeneous and multifactorial neurodegenerative disease, which is predicted to affect 112 million people worldwide in 2040 [1]. Primary open-angle glaucoma (POAG), as the most common type of glaucoma, is the second leading cause of blindness in the United States [2]. Although increased intraocular pressure (IOP) is a confirmed risk factor, the pathogenesis of glaucoma is not monofactorial. Several studies implicated vascular risk factors in the pathogenesis of glaucoma [3, 4]. The vascular theory considers glaucomatous optic neuropathy a consequence of insufficient ocular blood supply due to vascular dysregulation [5]; however, the mechanism of vascular dysregulation in glaucoma is largely unknown.

Platelet function and platelet-endothelial interactions are involved in the ocular microcirculation, with implications based on clinical and experimental evidence. Watanabe et al. [6] observed involvement of platelet coagulation and inflammation in the endothelium of Schlemm's Canal in POAG. Nishijima et al. [7] found that platelets actively interacted with retinal endothelial cells in the postischemic retina. It is well known that platelets are essential contributors to the blood coagulation and microvascular network. Platelet activation as well as their interaction with vascular endothelium and plasma components play an important role in vascular pathophysiology [8]. Platelets also express numerous surface receptors, which are involved in platelet cross talk with immune and endothelial cells under physiological and pathological conditions [9, 10]. Activated platelets change their shape, cytoskeletal structure, and mechanical properties. Some observed an altered platelet aggregation in glaucoma; moreover, increased platelet aggregation has a negative influence on blood flow in the small branches of the short ciliary arteries supplying the optic disk [11]. Measuring platelet parameters, which can partially imply platelet function and activation, has been widely used to evaluate disease development and progression [12, 13]. Our previous study reported that mean platelet distribution width (PDW) and mean platelet volume (MPV), both of which are markers of platelet activation, were increased in patients with neovascular glaucoma and primary angle-closure glaucoma [14, 15]. To our knowledge, no previous study has investigated the association between platelet parameter and POAG.

To better understand platelet activation in POAG, we performed a retrospective study to measure platelet parameters in patients with POAG and to explore a possible association between platelet parameters and POAG severity.

## 2. Materials and Methods

This study was approved by the Ethics Review Committee of Eye and Ear, Nose, Throat Hospital (EENT), Fudan University. The design and implement of this study adhered to the tenets of the Declaration of Helsinki. Written informed consent was obtained from all the patients.

### 2.1. Patients

Patients were consecutively recruited in the Eye and ENT Hospital, Fudan University, from January 2016 to October 2018. A total of 520 participants were recruited, of whom 118 (secondary glaucoma = 22, congenital glaucoma = 8, other concomitant eye diseases = 19, normal tension glaucoma = 11, receive antiplatelets/anticoagulants medications mentioned in exclusion criteria = 12, renal diseases = 8, hematological diseases = 1, thyroid dysfunction = 9, cardiovascular diseases = 10, hepatic diseases = 8, acute infectious diseases = 3, autoimmune diseases = 6, and cancer = 1) were later excluded, leaving a final sample of 402 patients.

The definition of POAG was based on (1) glaucomatous optic neuropathy such as a vertical cup disk ratio (VCDR) > 0.7 or an inter-eye asymmetry of >0.2, with notching, rim thinning, or retina nerve fiber layer (RNFL) defect; (2) visual field defect that corresponds to the structural change: presence of at least three contiguous nonedged test points within the same hemifield on the corrected probability plot at *p* < 0.05, with at least one point *p* < 0.01, excluding points directly above and below the blind spot; (3) the anterior chamber angle was considered open and normal in appearance on gonioscopy for both eyes [16].

Excluded from this study were patients with congenital, secondary glaucoma or history of intraocular surgery. Patients with concomitant ocular diseases, which could potentially impair visual fields such as optic disk anomalies, optic nerve diseases, retinal diseases, pathologic myopia, and intracranial lesions, were excluded. A review of systemic diseases was conducted, and patients who met the following criteria were also excluded: (1) <18 years old or pregnant woman; (2) hematological diseases such as aplastic anaemia, purpura haemorrhagica, and primary thrombocytosis; (3) abnormal coagulation function; (4) severe cardiovascular, hepatic, or renal diseases; (5) recent surgery or trauma; (6) cancer; (7) acute infectious diseases or autoimmune diseases; (8) thyroid dysfunction; (9) use of antiplatelets/anticoagulants medications during the previous 6 months such as aspirin, clopidogrel, warfarin, and cilostazol [17].

### 2.2. Control Subjects

Healthy controls were consecutively recruited from individuals who participated in yearly health screenings during the study period. A total of 500 individuals were recruited, of whom 92 (receive antiplatelets/anticoagulants medications = 21, eye diseases = 15, IOP ≥ 21 mmHg = 4, VCDR > 0.5 = 7, thyroid dysfunction = 10, cardiovascular diseases = 15, hepatic diseases = 6, renal diseases = 3, acute infectious diseases = 2, autoimmune diseases = 4, recent surgery = 2, and cancer = 3) were later excluded from the study according to the inclusion criteria, leaving a final sample of 408 control subjects.

Inclusion criteria: IOP < 21 mmHg; age 18 years and older; normal-appearing optic discs; anterior chamber angle open; VCDR ≤ 0.5. Exclusion criteria: family history or personal history of glaucoma; complaints of eye discomfort; prior ocular trauma or surgery; severe systemic diseases.

### 2.3. Clinical Examination

All the enrolled POAG subjects in the study were inpatients, and blood samples were drawn for laboratory measurements at the same day that standardized ophthalmic examination and comprehensive physical examination were performed. Each subject underwent a thorough ophthalmological examination conducted by a glaucoma specialist. Gonioscopy was performed for all the recruited patients, to determine anterior chamber angle. Visual fields mean deviation (MD) and visual fields mean sensitivity (MS) were measured with the Octopus automated perimeter (HAAG, STREIT, Switzerland). The intraocular pressure (IOP) was measured 3 times with a Goldmann applanation tonometer, and the average value was determined. Fundus photography was performed using a digital retinal camera (TRC- NW200, Topcon). Each control individual underwent preliminary ophthalmic examinations, which included refractive status, gonioscopy, slit-lamp biomicroscopic examination, and IOP, as carried out by glaucoma specialists. Clinical and demographic information was obtained from the medical data platform of the hospital. The subjects' drinking (>3 times per week and >6 mo (current or former)) and smoking (>1 cigarette per day and >6 mo (current or former)) history (self-reported) were also collected.

Laboratory measurements were performed in the Department of Clinical Laboratory Science, Eye & ENT Hospital. Blood samples for platelet parameters, including platelet count (PLT), platelet distribution width (PDW), plateletcrit (PCT), mean platelet volume (MPV), and platelet large cell ratio (P-LCR), were taken in laboratory tubes with ethylenediaminetetraacetic acid (EDTA) and analyzed using an automated hematology analyzer (Sysmex XN 1000, Japan) within 30 minutes following standard venipuncture of the veins in the antecubital fossae (anterior elbow veins). The reference range of PLT, MPV, PDW, PCT, and P-LCR was (100–400) *∗* 10^9^/L, 9.0–16.0 fL, 9.0–17.0 fL, 0.16–0.22%, and 14.0–46.0%, respectively. Quality control of the automated hematology analyzer was performed each day before sample detection with three levels (low, medium, and high) of quality control materials. Internal laboratory quality controls were analyzed daily over a 3-year period, with no significant changes in the values.

### 2.4. Subgroup Analysis

POAG has a male predominance [18]. Moreover, there was greater number of males than females in POAG participants (268 *vs*. 134) in the present study. This is consistent with our study in 2 previous studies [19, 20] that men constituted 60.6% and 83.8%, respectively, of POAG patients. Therefore, the participants were categorized into gender subgroups. The prevalence of glaucoma increased with age [21]. Therefore, within the male and female subgroups, the POAG patients were further subdivided into 2 groups based on age: a <50 subgroup and a ≥50 subgroup. POAG patients were then further subdivided into 3 different groups based on the disease severity according to their MD results as follows: mild (MD ≤ 6.00 dB), moderate (6 < MD ≤ 12 dB), and severe (MD > 12 dB) POAG [22]. Because self-detection of glaucoma by affected individuals usually occurs at a late stage of the disease, most of the glaucoma patients in China paid too little attention to their eye discomfort until they subjectively felt vision deterioration. Therefore, most of the glaucoma patients who presented to the hospital were at the severe stage [23].

### 2.5. Statistical Analyses

The data were analyzed by SPSS23.0 (IBM SPSS Statistics) and Microsoft Excel 2016. The results are presented as mean ± standard deviation (SD). The independent Student's *t*-test and *χ*^2^ test were used for the comparison of participant characteristics between the groups. A one-way analysis of variance was used to compare the platelet and ocular parameters among the 3 disease-severity subgroups. The associations between platelet parameters and ocular parameters in POAG were analyzed using Spearman correlation, after which multivariate linear regression analyses were performed to evaluate the association between platelet parameters and disease severity, using MD, VCDR, and MS. Logistic regression analyses were performed to identify the association between platelet parameters and severity of POAG (mild and moderate POAG group = 1; severe POAG group = 2) (male = 1, female = 2; no hypertension = 1, hypertension = 2; no diabetes = 1, diabetes = 2; no drinking = 1, drinking = 2; no smoking = 1, smoking = 2). Odds ratios (ORs) with 95% confidence intervals (95% CIs) were estimated by logistic regression analyses. A two-sided *p* value of <0.05 was considered statistically significant.

## 3. Results

### 3.1. Demographic and Platelet Parameters of the Study Subjects

A total of 402 POAG patients (male = 268; female = 134) and 408 control subjects (male = 258; female = 150) were enrolled. Their mean ages were 51.19 ± 15.69 years and 51.50 ± 8.90 years in POAG and control groups, respectively. If both eyes of the same individual were affected by POAG, only one eye was randomly selected. The POAG group and control group were closely matched in terms of mean age (*p*=0.733) and gender (*p*=0.338). Hypertension (*p*=0.107), diabetes (*p*=0.611), smoking (*p*=0.550), and alcohol consumption (*p*=0.580) status did not show statistical differences between the two groups.  Table 1 summarized the demographic and clinical characteristics of the participants.

Table 1 also showed comparison of platelet parameters in the POAG and control group. POAG patients had significantly lower (*p*=0.001) PLT levels (207.08 ± 54.70 *∗* 10^9^/L) than those of the control group (220.46 ± 55.85 *∗* 10^9^/L). In the POAG group, the PDW (13.76 ± 3.16 fL) and MPV (10.46 ± 1.32 fL) values were significantly higher (all *p* < 0.001) than those of the control group (PDW 11.82 ± 2.44 fL, MPV 10.13 ± 1.10 fL). Patients with POAG (0.21 ± 0.05%) had slightly lower (*p*=0.045) PCT than the control group (0.22 ± 0.05%). No significant difference was found between the two groups regarding P-LCR (*p*=0.056).

### 3.2. Comparison of Platelet Parameters in Subjects with POAG Stratified according to Gender and Age

According to their age and gender, POAG and control subjects were divided into male (a <50 subgroup, and a ≥50 subgroup) and female subgroups (a <50 subgroup and a ≥50 subgroup). In both the gender and age subgroups, the PDW and MPV levels were significantly higher (all *p* < 0.05) in the POAG group in comparison to the control group (Table 2). There were no significant differences between the two groups regarding PCT and P-LCR in all subgroups (*p* > 0.05). The decreasing changing trend of PLT was similar in all subgroups of POAG patients (*p* < 0.05) compared to the control group, although the change was not statistically significant in female subjects who were younger than 50 (Table 2).

### 3.3. Comparison of Demographics, Platelet Parameters, and Ocular Parameters in Subjects with POAG, Stratified according to Severity

**Figure 1:**
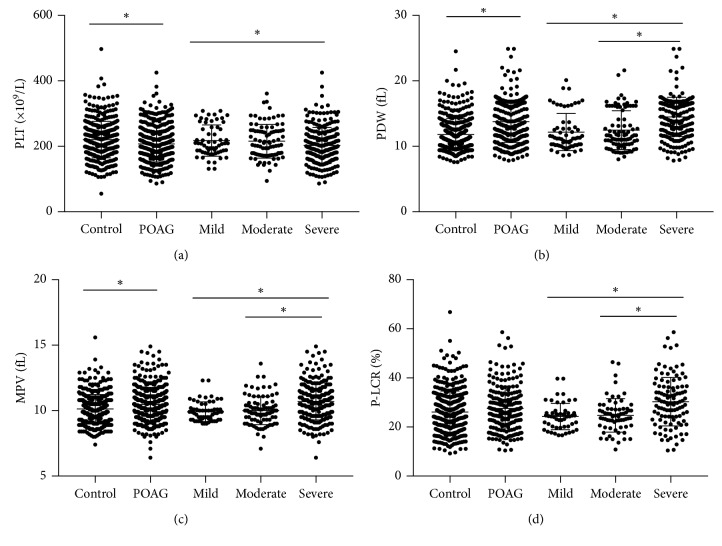
Comparison of (a) platelet (PLT), (b) platelet distribution width (PDW), (c) mean platelet volume (MPV), and (d) platelet large cell ratio (P-LCR) levels in patients with mild, moderate, and severe primary open-angle glaucoma (POAG) and the control group. Each data point represents one subject. The medium bar represents the mean; the top and bottom bars represent the standard deviation. ^*∗*^Statistical significance (*p* < 0.05) between the two groups.

On the basis of the MD, the POAG subjects were categorized into 3 subgroups of different disease-severity levels, whereby 70 subjects were classified as mild, 94 as moderate, and 238 as severe. A comparison of platelet parameters and ocular parameters in the POAG subjects is shown in Table 3 and Figure 1. No statistical differences in gender (*p*=0.313) and age (*p*=0.882) among the three groups were observed. The PDW and MPV were highest in the severe POAG group (PDW 14.49 ± 2.99 fL; MPV 10.74 ± 1.39 fL), followed by the moderate POAG group (PDW 12.50 ± 3.14 fL; MPV 10.02 ± 1.08 fL) and then the mild POAG group (PDW 11.82 ± 2.44 fL; MPV 9.92 ± 0.76 fL), with statistically significant differences observed between mild-severe POAG and moderate-severe POAG by LSD post hoc test (*p* < 0.05). P-LCR also had significant differences between the disease-severity subgroups, although no significant difference was found between the POAG and control group. The PLT was significantly lower (*p* < 0.05) in severe POAG (200.77 ± 52.18 *∗* 10^9^/L) than moderate POAG (218.16 ± 54.98 *∗* 10^9^/L). The detailed information is shown in Table 3.

### 3.4. Spearman Correlation and Multiple Linear Regressions for Associations between Platelet Parameters and Ocular Parameters in Patients with POAG

**Figure 2:**
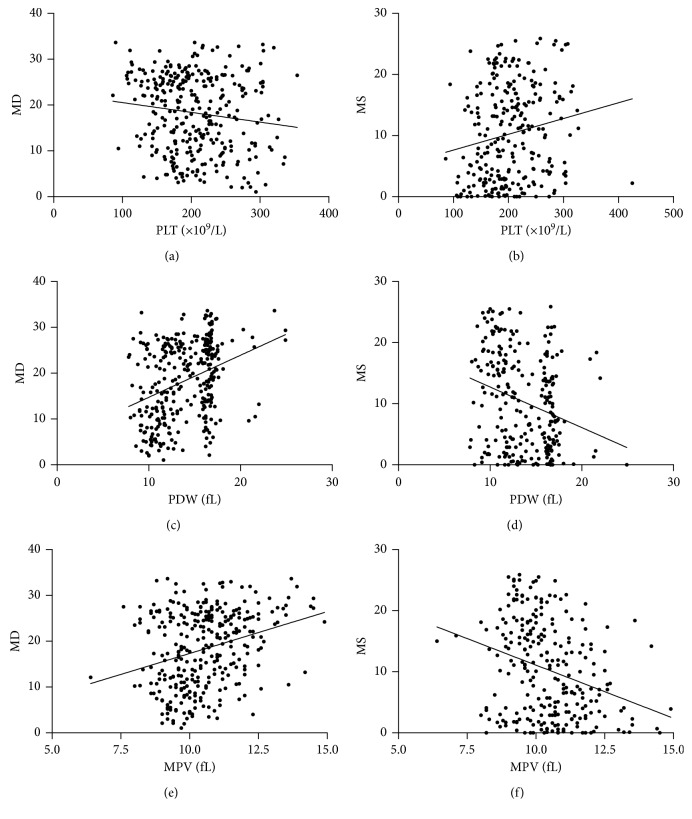
Scatterplot of patient individual measurements for visual field median deviation (MD) and visual field mean sensitivity (MS) versus platelet (PLT), platelet distribution width (PDW) and mean platelet volume (MPV) levels; each data point represents one patient. Linear regression is displayed.

Spearman correlation analyses were performed to identify the association between platelet parameters and ocular parameters in patients with POAG (Table 4 and Figure 2). There was a statistically significant correlation between PLT, PDW, MPV, and P-LCR levels and MD and MS. After spearman analyses, a multiple linear regression analysis adjusting for age, gender, BMI, hypertension, diabetes, drinking, and smoking was performed to further analyze the association between platelet parameters and ocular parameters in POAG (Table 5). There was a statistically significant association between PDW and MD (*β* = 0.430, *p* < 0.001); PDW and MS (*β* = −0.317, *p*=0.009); MPV and MD (*β* = 0.363, *p*=0.001); and MPV and MS (*β* = −0.359, *p*=0.003) in POAG patients.

### 3.5. Logistic Regression Analysis of the Association between Platelet Parameters and Severity of POAG

Logistic regression analyses were performed to identify the association between platelet parameters and severity of POAG (Table 6). Logistic regression analyses revealed that PDW (OR = 1.297, 95% CI = 1.011–1.663) and BMI (OR = 1.195, 95% CI = 1.014–1.409) were associated with severity of POAG.

## 4. Discussion

To the best of our knowledge, this is the first study to evaluate the potential relationship between platelet parameters and POAG. Our results indicated that POAG patients had significantly lower platelet counts and significantly higher PDW and MPV levels than control subjects. The PDW and MPV levels were highest in the severe POAG group, followed by the moderate POAG group and then the mild POAG group. Logistic regression analyses revealed that PDW was associated with the severity of POAG. Our results suggest altered platelet activation in POAG patients.

Platelets play a vital role in the coagulation cascade and in vascular pathophysiology [24]. MPV shows the average platelet volume in the blood, while PDW reflects the heterogeneity in platelet volume. Both MPV and PDW are markers of platelet activation. In our present study, we found that in overall POAG patients, both the gender and age subgroups of POAG, the PDW and MPV levels were all significantly higher (all *p* < 0.05) in comparison to those of the control group. Larger platelets are metabolically, enzymatically more active [25] and have greater prothrombotic potential [26]; they produce more glycoprotein Ib and glycoprotein IIb/IIIa receptors, release more thromboxane A2 and rapidly aggregate [27]. Several studies have found an altered platelet aggregation in POAG. Hoyng et al. [28] found an age-dependent association between spontaneous platelet aggregation (SPA) and the presence of POAG. Matsumoto et al. [29] also confirmed the high prevalence of increased platelet aggregation in patients with normal tension glaucoma and POAG. Although the pathogenetic role of altered platelet aggregation is not yet clear, it has been assumed that the increased platelet aggregation has a negative influence on the blood flow in the small branches of the short ciliary arteries supplying the optic disk [11]. In addition, evidence suggests platelet activation plays an important role in the pathogenesis of ischemia-reperfusion injury [30]. Fluctuation of blood flow due to abnormal vascular regulation may lead to ocular ischemia-reperfusion injury in glaucoma patients. Nishijima et al. [7] have demonstrated that platelets actively interacted with retinal endothelial cells in the postischemic retina through P-selectin expressed on the retinal endothelial cells.

Ocular blood flow alterations in glaucoma patients seem, at least partly, to be associated with systemic vascular dysregulation. Platelet parameters, especially platelet activation parameters, have been reported to be related to several ocular diseases and cardiovascular event. T. Yilmaz and A. Yilmaz [31] found that an increased proportion of large platelets (a higher MPV, PDW, and P-LCR) was a risk factor for developing retinal vein occlusion. Citirik et al. [32] observed a higher MPV value in diabetic retinopathy patients compared with healthy subjects and a gradual increase in MPV values with increasing severity of diabetic retinopathy. In atherosclerosis, platelets contribute to the endothelial dysfunction as well as the rupture of the vulnerable plaque [33]. The interaction of platelets with endothelial cells leads to excessive platelet activation, thus results in shorter half-life and increased platelet turnover, which was reflected in the PLT, PDW, MPV, and P-LCR levels.

Reduced ocular blood flow is related to the progression of visual field loss in glaucoma [34]. In the present study, we also found that PDW, MPV, and P-LCR were increased and positively correlated with POAG severity. Hoyng et al. [35] reported that the percentage patients with spontaneous platelet aggregation were higher for POAG patients with visual field deterioration (60%) than POAG patients without progressive loss of visual fields (12.5%, *p* < 0.005). In Alzheimer's disease, which is a neurodegenerative disease, vascular risk factors also contribute to the progression of dementia and influence platelet activation. Stellos et al. [36] reported that a significantly higher expression of platelet activation biomarkers was observed in patients with AD with fast cognitive decline compared with AD patients with slow cognitive decline. Based on the above findings, we speculate that platelet activation may exacerbate the reduction of ocular blood flow and play an important role in the disease progression, especially in severe POAG patients.

Logistic regression analyses revealed that PDW was independently associated with severity of POAG. PDW is a more direct marker to represent platelet reactivity than MPV since it was not elevated during single platelet distention caused by platelet swelling [37, 38]. These findings, along with the above results, suggested altered platelet activation in POAG and its development. Logistic regression analyses also revealed that BMI was a risk factor for severe POAG, which was consistent with the previous study [39]. In the present study, we observed that POAG patients had significantly lower PLT counts, however, regression analyses showed that PLT was not independently associated with POAG severity. We speculate that the platelet function, rather than the numbers of platelets, is more related to the pathophysiology of POAG.

The platelet parameter values of most patients with POAG were still within the reference range, thus we consider platelet function may not be the primary cause of POAG but may be a secondary factor that could increase the risk of POAG and be involved in disease development. However, our results might provide a vital novel field for researchers to study the pathophysiological mechanism of POAG. Considering that vascular dysregulation is commonly recognized to be involved in glaucoma, it is worth exploring the possible mechanism of vascular dysregulation in glaucoma. Moreover, platelet activation, either secondary to vascular dysregulation or resulting in vascular dysregulation, can be well monitored in a clinical laboratory.

### 4.1. Limitation

We acknowledge that our present study has some limitations. Firstly, our study was a single-center, retrospective analysis. The results might be affected by confounding factors, despite that multiple linear regression analysis was performed to adjust for age, gender, BMI, hypertension, diabetes, smoking, and drinking status. Therefore, large-scale, multi-center prospective studies are required to better investigate the relationship between platelet parameters and POAG. Secondly, due to the inherent deficiencies of retrospective methods, we cannot judge the causality. The mechanism of platelet activation in the pathogenesis of glaucomatous optic neuropathy is worth further exploration in our follow-up study. Moreover, visual field testing was not performed in healthy controls since it is not a routine eye examination in our hospital, although each control individual underwent preliminary ophthalmic examinations.

To the best of our knowledge, this is the first study to assess a potential relationship between platelet parameters and POAG. We found that POAG patients have higher PDW, which has a significant positive correlation with POAG severity. Our results provide further evidence of the vascular dysregulation in POAG, raising the possibility of modulating platelet activation as a potential therapeutic measure.

## Figures and Tables

**Table 1:** Demographics and platelet parameters of subjects with POAG.

	POAG (*n*=402)	Control (*n*=408)	*T*- or chi-square value	*p* value
Age (y)	51.19 ± 15.69	51.50 ± 8.90	−0.341	0.733
Female/male	134/268	150/258	1.047	0.338
BMI	24.31 ± 3.47	24.13 ± 3.38	0.426	0.670
Hypertension (yes/no)	75/327	58/350	2.910	0.107
Diabetes (yes/no)	31/371	36/372	0.330	0.611
Smoking (yes/no)	62/340	56/352	0.469	0.550
Drinking (yes/no)	73/329	68/340	0.314	0.580
PLT (10^9^/L)	207.08 ± 54.70	220.46 ± 55.85	−3.392	**0.001**
PDW (fL)	13.76 ± 3.16	11.82 ± 2.44	9.571	**<0.001**
PCT (%)	0.21 ± 0.05	0.22 ± 0.05	−2.009	**0.045**
MPV (fL)	10.46 ± 1.32	10.13 ± 1.10	3.810	**<0.001**
P-LCR (%)	27.57 ± 9.14	26.13 ± 8.50	1.915	0.056

Independent-samples *T*-test and *χ*^2^ test were used. Data are expressed as mean ± SD. POAG: primary open-angle glaucoma; BMI: body mass index; PLT: platelet count; PDW: platelet distribution width; PCT: plateletcrit; MPV: mean platelet volume; P-LCR: platelet large cell ratio.

**Table 2:** Comparison of platelet parameters in subjects with POAG, stratified according to gender and age.

Subgroups	POAG (*n*=402)	Control (*n*=408)	*T*-value	*p* value
*PLT (10* ^*9*^ */L)*				
Male				
<50 (*n*=131, 129)	210.18 ± 52.23	227.53 ± 53.51	−2.614	**0.009**
≥50 (*n*=137, 129)	190.62 ± 47.83	203.81 ± 51.53	−2.107	**0.036**
Female				
<50 (*n*=52, 69)	227.19 ± 58.51	234.32 ± 53.53	−0.671	0.504
≥50 (*n*=82, 81)	206.73 ± 48.66	222.64 ± 48.95	−2.048	**0.042**
*PDW (fL)*				
Male				
<50	13.95 ± 2.99	11.70 ± 2.30	6.712	**<0.001**
≥50	13.63 ± 3.28	11.76 ± 2.42	5.190	**<0.001**
Female				
<50	13.84 ± 3.52	12.40 ± 2.80	2.349	**0.021**
≥50	13.63 ± 3.05	11.63 ± 2.35	4.639	**<0.001**
*PCT (%)*				
Male				
<50	0.22 ± 0.04	0.23 ± 0.05	−1.373	0.171
≥50	0.20 ± 0.05	0.20 ± 0.05	−0.601	0.548
Female				
<50	0.24 ± 0.06	0.24 ± 0.06	−0.433	0.666
≥50	0.21 ± 0.05	0.22 ± 0.04	2.293	0.296
*MPV (fL)*				
Male				
<50	10.54 ± 1.38	10.09 ± 1.01	2.921	**0.004**
≥50	10.40 ± 1.34	10.07 ± 1.05	2.207	**0.028**
Female				
<50	10.68 ± 1.20	10.23 ± 1.00	2.151	**0.034**
≥50	10.47 ± 1.14	10.06 ± 1.13	2.293	**0.023**
*P-LCR (%)*				
Male				
<50	27.67 ± 9.22	25.66 ± 8.10	1.530	0.128
≥50	27.65 ± 8.52	25.70 ± 8.18	1.585	0.114
Female				
<50	26.42 ± 9.67	28.25 ± 9.50	−0.848	0.399
≥50	27.99 ± 9.88	25.81 ± 8.64	1.280	0.203

Independent-samples *T*-test and *χ*^2^ test were used. Data are expressed as mean ± SD. POAG: primary open-angle glaucoma; PLT: platelet count; PDW: platelet distribution width; PCT: plateletcrit; MPV: mean platelet volume; P-LCR: platelet large cell ratio.

**Table 3:** Comparison of demographics, platelet parameters, and ocular parameters in subjects with POAG, stratified according to severity.

Factors	Mild POAG (*n*=70)	Moderate POAG (*n*=94)	Severe POAG (*n*=238)	*p* value
Age (y)	50.53 ± 16.00	51.83 ± 15.60	50.74 ± 15.66	0.882
Female/male	24/46	37/57	73/165	0.313
BMI	23.21 ± 2.79	23.58 ± 2.91	24.92 ± 3.68	**0.048** ^b^
IOP (mm·Hg)	19.30 ± 6.60	20.43 ± 8.53	25.74 ± 11.21	**<0.001** ^bc^
VCDR	0.60 ± 0.20	0.71 ± 0.19	0.87 ± 0.13	**<0.001** ^abc^
MD (dB)	4.13 ± 1.28	9.34 ± 1.61	22.45 ± 5.53	**<0.001** ^abc^
MS (dB)	23.39 ± 1.45	18.25 ± 1.87	6.24 ± 4.95	**<0.001** ^abc^
PLT (10^9^/L)	217.43 ± 48.11	218.16 ± 54.98	200.77 ± 52.18	**0.036** ^c^
PDW (fL)	11.82 ± 2.44	12.50 ± 3.14	14.49 ± 2.99	**<0.001** ^bc^
PCT (%)	0.21 ± 0.04	0.22 ± 0.05	0.21 ± 0.05	0.744
MPV (fL)	9.92 ± 0.76	10.02 ± 1.08	10.74 ± 1.39	**<0.001** ^bc^
P-LCR (%)	24.35 ± 5.90	24.58 ± 7.41	30.12 ± 9.71	**0.001** ^bc^

*χ* ^2^ test and 1-way analysis of variance (ANOVA) were used. Data are expressed as mean ± SD. ^a^*p* < 0.05 for the difference between Mild POAG and Moderate POAG (1-way ANOVA with the LSD post hoc test). ^b^*p* < 0.05 for the difference between Mild POAG and Severe POAG (1-way ANOVA with the LSD post hoc test). ^c^*p* < 0.05 for the difference between Moderate POAG and Severe POAG (1-way ANOVA with the LSD post hoc test). POAG: primary open-angle glaucoma; BMI: body mass index; IOP, intraocular pressure; VCDR, vertical cup-disc ratio; MD, visual field mean deviation; MS, visual fields mean sensitivity; PLT: platelet count; PDW: platelet distribution width; PCT: plateletcrit; MPV: mean platelet volume; P-LCR: platelet large cell ratio.

**Table 4:** Correlation between platelet parameters and glaucoma severity in POAG.

Factors	IOP	VCDR	MD	MS
PLT	*r* = 0.029, *p*=0.576	*r* = −0.080, *p*=0.126	*r* = −0.129, **p=0.022**	*r* = 0.197, **p=0.002**
PDW	*r* = 0.011, *p*=0.832	*r* = 0.106, **p=0.042**	*r* = 0.350, **p<0.001**	*r* = −0.266, **p<0.001**
MPV	*r* = 0.069, *p*=0.186	*r* = 0.079, *p*=0.133	*r* = 0.283, **p<0.001**	*r* = −0.298, **p<0.001**
P-LCR	*r* = 0.085, *p*=0.229	*r* = 0.020, *p*=0.777	*r* = 0.306, **p<0.001**	*r* = −0.263, **p=0.002**
PCT	*r* = 0.028, *p*=0.595	*r* = −0.072, *p*=0.172	*r* = −0.035, *p*=0.541	*r* = 0.095, *p*=0.132

POAG: primary open-angle glaucoma; PLT: platelet count; PDW: platelet distribution width; PCT: plateletcrit; MPV: mean platelet volume; P-LCR: platelet large cell ratio; IOP, intraocular pressure; VCDR, vertical cup-disc ratio; MD, visual field mean deviation; MS, visual fields mean sensitivity. One-sample Kolmogorov–Smirnov test *P* < 0.05. Spearman correlation was used.

**Table 5:** Multiple linear regressions for associations between platelet parameters and ocular parameters in patients with POAG.

	VCDR *β* (*p*, 95% CI)	MD *β* (*p*, 95% CI)	MS *β* (*p*, 95% CI)
PLT	−0.166 (0.097, −98.50 to 8.39)	−0.182 (0.073, −1.982 to 0.092)	0.173 (0.161, −0.377 to 2.228)
PDW	0.145 (0.167, −1.136 to 6.450)	0.430 (<0.001, 0.083 to 0.218)	−0.317 (0.009, −0.191 to −0.028)
MPV	0.209 (0.050, 0.003 to 3.023)	0.363 (0.001, 0.022 to 0.078)	−0.359 (0.003, −0.081 to −0.017)

Adjusting for age, gender, body mass index, hypertension, diabetes, drinking, and smoking. 95% CI: 95% confidence interval; POAG: primary open-angle glaucoma; PLT: platelet count; PDW: platelet distribution width; PCT: plateletcrit; MPV: mean platelet volume; P-LCR: platelet large cell ratio; VCDR: vertical cup-disc ratio; MD: visual fields mean deviation; MS: visual fields mean sensitivity.

**Table 6:** Logistic regression analysis of the association between platelet parameters and severity of POAG.

	OR	*p* value	95% CI
Age	1.012	0.585	0.971–1.054
Gender	0.820	0.709	0.289–2.326
BMI	1.195	**0.034**	1.014–1.409
Hypertension	2.403	0.309	0.444–13.018
Diabetes	2.560	0.287	0.454–14.449
Smoking	1.105	0.881	0.299–4.079
Drinking	1.380	0.588	0.430–4.435
PDW	1.297	**0.040**	1.011–1.663
MPV	1.055	0.862	0.574–1.940
PLT	0.995	0.431	0.984–1.007

Logistic regression analyses were performed to identify the association between platelet parameters and severity of primary open-angle glaucoma (POAG) (mild and moderate POAG group = 1; severe POAG group = 2) (male = 1, female = 2; no hypertension = 1, hypertension = 2; no diabetes = 1, diabetes = 2; no drinking = 1, drinking = 2; no smoking = 1, smoking = 2). 95% CI: 95% confidence interval; POAG: primary open-angle glaucoma; BMI: body mass index; PLT: platelet count; PDW: platelet distribution width; MPV: mean platelet volume.

## Data Availability

The data used to support the findings of this study are available from the corresponding author upon request.
